# Diuretic Medication in Various Diseases

**Published:** 1902-12

**Authors:** T. Solacolu

**Affiliations:** Paris


ATLANTA

### Journal-Record of Medicine.

Successor to Atlanta Medical and Surgical Journal, Established 1855,
and Southern Medical Record, Established 1870.

VOL. IV. DECEMBER, 1902. No. 9.

BERNARD WOLFF, M.D., EDITOR, Nos. 319–20 Prudential.

Published Monthly.

M. B. HUTCHINS, M.D., BUSINESS MANAGER, No. 64 Marietta St.

### *ORIGINAL COMMUNICATIONS.*

### DIURETIC MEDICATION IN VARIOUS DISEASES.

BY T. SOLACOLU, M.D.,
PARIS.

Among the diuretics which comprise our therapeutic armamentarium there is none that is beyond criticism. After many trials a medicament which always promoted an abundant diuresis may fail completely in an emergency. Moreover, diuretics which often act well in anuria or oliguria of cardiac origin may be inefficient in those of renal origin, and finally the solubility or nonsolubility of the drug plays an important part in its physiological action. Thus, for instance, digitalis, which is certainly one of the most precious drugs in the dropsy of cardiac origin, is usually inefficient in that due solely to impaired function of the kidneys. We cannot do better than to quote from Huchard, whose competence on this question is incontestable:

"During a certain part of the disease, in which both heart and kidneys are affected, the renal factor is more prominent than the cardiac, that is to say, the renal insufficiency predominates over the cardiac insufficiency. Then an exclusive milk diet and theobromine, which has no action upon the heart and whose effects are concentrated upon the kidneys, act marvelously for some time. After this we notice an edema of another character manifest itself, and we are astonished that the same medication no longer produces the same effect. The patient who has been a renal subject for several days shows himself now as a cardiac subject, and the edema of the renal type of several days ago now has a cardiac origin. Hence, theobromine, which acts particularly upon the kidneys and but little on the heart, now becomes an unreliable diuretic and the time for digitalis has come."

Digitalis and theobromine are two excellent diuretics, but the latter has the inconvenience of dissolving with great difficulty. Lately our attention has been drawn to a communication of a Belgian physician, Professor Destree, presented to the Therapeutic Society of Paris, upon a soluble salt of theobromine, the double acetate of theobromine sodium, or agurine.

Under the direction of Dr. Lancereaux, who has wished me to make a special study, we have instituted a series of clinical observations upon the different patients suffering with cardiac or renal diseases, and it is the result of these observations that seems to us of interest to relate here.

Before commencing the clinical study of this drug I think it would be useful to give a brief résumé of its chemical and physical characters. Theobromine is a derivative of the xanthin series, and is insoluble in water. Gram prepared a soluble double salt of theobromine sodium and salicylate of sodium, which is very soluble in water and is not caustic. Agurin, which was prepared by Dr. Impens, of Brussels, is a double salt of theobromine sodium and acetate of sodium, which is soluble in water and appears in the form of a white hygroscopic powder. Its reaction is strongly alkaline, it contains about 60 per cent. of theobromine, and is not caustic. Owing to its solubility it is readily taken up and exerts its diuretic influence within twenty-four hours after its administration. When subcutaneously injected in animals it may produce local induration at the points of injection, but not abscesses. For this reason it is not adapted for this method of employment. The effects produced by the drug after its absorption are as follows: Upon the heart we have not noticed any particular influence, while the arterial tension is slightly increased. The effects upon the nervous system were nil, and no headache or excitement were noticed. The principle action of the drug is upon the kidneys, the urinary secretion being augmented, not only the quantity but the solids being increased. From the observations made by Destree and myself, it may be concluded that the mode of action of agurin closely resembles that of theobromine, both acting directly upon the renal epithelium. According to the classification of Hayen, theobromine belongs among the direct diuretics which act by exciting the function of the renal epithelium. Contrary to the opinion of Villegeane, theobromine is not eliminated as such, but according to the experiments of Boudzinsk and Gottlieb, it is transformed in the organism into methyl xanthin. A certain part of the theobromine is eliminated in the urine as such; a second part is oxidized; a third part is eliminated as paraxanthine, and finally, a fourth part is converted into trimethyl xanthin. Acetate of sodium, which also enters into the composition of agurin, is transformed and eliminated as the carbonate of sodium.

To determine the toxicity of agurin we have made a series of experiments on rabbits, injecting a 25 per cent. solution subcutaneously. Injection of 1 c.cm. of a 25 per cent. solution into a rabbit weighing 1200 gm. produced some acceleration of the respiration and pulse. Injection 4 c.cm. of the same solution into a rabbit weighing 1800 gm. caused at first acceleration of pulse and respiration, followed by stiffness of the limbs, convulsions and death. Injection of 5 c.cm. of the same solution into a rabbit weighing 1400 gm. produced convulsions and death at the end of injection. Injection of 3 c.cm. into a rabbit weighing 1800 gm. followed six minutes later by another injection of the same amount. After first injection acceleration of respiration and pulse lasting three or four minutes, but no convulsions; after second injection the same effect but less marked.

Next we shall study the clinical results obtained from the use of agurin. The drug has been employed by us in cases in which theobromine has been indicated. The clinical material may be divided into three classes: 1. Arteriosclerosis of the heart, arrhythmia, edema, but without renal lesion. 2. Arteriosclerosis of the kidneys with hypertrophy of the heart. 3. Peritoneal effusion of hepatic origin and pleural effusion.

In the first class of cases there were present very marked arterial lesions, cardiac pulsations were feeble and arrhythmic, but the limbs very little edematous. The patients suffered with oppression and dyspnea on exertion. The urine contained little or no albumin. In general, the quantity of urine secreted ranged from 250 to 500 c.cm.; but three or four days after the administration of agurin it rose to 2500, 3000, and 3500 c.cm. The specific gravity of the urine was increased. Following the diuresis the edema diminished; the feeling of oppression disappeared, and in two instances the heart action became regular.

In the second class of cases of arteriosclerosis the kidneys were affected and the patients presented symptoms of uremia. In cases of uremia of a light degree, with slight vomiting, nausea, slight headache, pains in the renal region, and a reduction of the quantity of urine below 500 c.cm., the use of agurin in solution or wafers, and in comparatively small doses, produced a very abundant diuresis with rapid improvement, which persisted during this treatment. In other cases of marked uremia with violent pains in the head preventing sleep, frequent bilious vomiting, malaise, the patients received agurin by rectal administration owing to their being unable to retain anything on their stomach. The enema contained 5 grains of agurin, 12 drops of laudanum, and 125 c.cm. of water. Under the use of the drug the urine, which had been very much reduced, ranging between 100 to 300 c.cm., rose to 1000 to 1200 c.cm. The headache was first relieved, but the vomiting was more persistent, and did not disappear completely until the end of about 6 to 10 days. In cases of peritoneal effusion in persons suffering with cirrhosis of the liver we obtained only very slight diuresis. We did not long continue the administration of the drug, and it is impossible to say whether it exerts any influence upon the cause of the effusion. In a case of pleurisy with effusion the diuresis also was insignificant.

In all our observations except one we failed to observe any signs of intolerance of the drug. In several cases we administered the remedy for one and even two weeks in daily doses of 3 to 4 gm., without observing any unpleasant by-effects. After its discontinuance the diuretic action usually persisted during a few days. In several instances we were compelled to renew its administration shortly after its suspension, and on each occasion it produced abundant diuresis.

CASE I.—M. A., aged thirty-five years, plumber; general arteriosclerosis; cardiac and renal disorders; albuminuria and uremia. The patient had lost his appetite, felt weak, and had been obliged to give up work on account of the feeling of oppression. Lungs normal; area of cardiac dulness increased; heart sounds accentuated; radial arteries hard and cordlike; no ascites or edema of the limbs. January 10, the quantity of urine fell from 2000 c.cm. to 500 c.cm., but after the administration of agurin, 1 gram, rose again to 3000 c.cm. It is difficult to state, however, whether the drug alone was responsible for this marked increase in the amount of urine.

CASE II.—C. B., twenty-eight years old, had suffered from chronic rheumatism for three years. In December, 1901, she suffered with palpitation of the heart, indigestion, and pain in the head. When admitted to the hospital, January, 1902, she complained of headache, pain in the renal region, nausea, vomiting. The patient was anemic; the face slightly puffed; the heart normal; stomach dilated; the urine showed traces of albumin. Under the use of agurin the quantity of urine was increased from 900 c.cm. to 1200 c.cm., later falling to 1000 c.cm. Symptoms of slight edema disappearing.

CASE III.—T. S., sixty years old, had suffered from frequent attacks of acute rheumatism. At the time of admission to the hospital she presented slight uremic symptoms; the quantity of urine was diminished. She suffered from headache, nausea, vomiting, pains in the kidney region. Examination showed the presence of albumin in the urine. Similar uremic seizures had occurred previously.

Under the administration of agurin the amount of urine was but slightly increased.

CASE IV.—B. J., aged fifty-eight years; arteriosclerosis, myocarditis, cardiac hypertrophy, emphysema, chronic bronchitis. For some time the patient had been greatly troubled with dyspnea on exertion, and at night was often compelled to sit up in bed, these symptoms having become severe during the past three months. No pain in the head; no vomiting. The quantity of urine had been reduced to 500 c.cm., in the 24 hours. Slight edema of the ankles. Under the use of agurin in doses of 1, 2, and 3 grams daily, an abundant diuresis was established. The amount of urine increased to 3500 c.cm. From May 17th to June 1st, it varied between 2500 and 1800 c.cm., and the feeling of oppression subsided. During the next four days the quantity fell to 1000 c.cm., and the patient again felt badly, so that the administration of agurin was resumed. On the third day the quantity of urine again reached 2500 c.cm., after which it again began to decrease.

CASE V.—T. E., thirty-five years old; arteriosclerosis; renal disease. The patient complained of a feeling of marked depression, especially at night. The limbs were swollen; the urine contained albumin. The administration of agurin in doses of 2 to 2.5 gm. daily increased the quantity of urine from 500 c.cm. to 1100 c.cm.

CASE VI.—M. L., fifty-one years old. Arteriosclerosis; myocarditis, asthma, chronic bronchitis. Since an attack of rheumatism at forty-two years of age the patient had never enjoyed good health, but suffered from dyspnea on exertion, and had to void his urine several times each night. His lips were bluish; the limbs slightly edematous, and the genitals markedly so. Under an absolute milk diet the quantity of urine increased but slightly and the edema became worse. On commencing the administration of agurin in 3.0 gm. doses daily the quantity of urine was considerably augmented, to 4½ liters, but diminished as soon as the drug was discontinued. During this time the swelling of the legs also subsided. When the drug was resumed at a later period the urinary quantity again became augmented.

CASE VII.—G. B., sixty-nine years old. Arteriosclerosis, myocarditis, renal disease. The patient complained of marked feeling of oppression; lips blue; heart enlarged; cardiac action irregular; arteries hard and sinuous; legs slightly swollen. Quantity of urine 250 c.cm. in the twenty-four hours. Trace of albumin. Under the administration of agurin, 4 gm. daily, the quantity of urine steadily increased, and on the third day had reached nearly two liters.

CASE VIII.—T. J., sixty-three years old. Cirrhosis of the liver; gradual loss of strength; inability to work; marked ascites and edema of the legs. The patient complained of severe oppression, and vomited anything that he took. Face congested; pupils dilated. Aspiration was resorted to, and three liters of fluid removed from the abdominal cavity. Owing to the risk of inducing vomiting agurin was given in amounts of 4 grams per rectum. The quantity of urine gradually increased to one liter, and its specific gravity also became augmented. Later the quantity varied between 1025 and 1030 c.cm.

CASE IX.—L. P., fifty-six years old; arteriosclerosis, myocarditis; marked ascites. The patient had taken théobromine on several occasions, but was unable to tolerate the drug on account of the disturbances in his stomach and vomiting. Agurin was given in doses of 0.5 gm. in wafers, and later in solution, but was not well borne in either form. It was then administered per rectum, but without success.

CASE X.—D. L., aged forty-seven years; lead poisoning; arteriosclerosis. The patient had had frequent attacks of lead colic and had presented uremic symptoms for about two years. When admitted to the hospital he suffered with a feeling of marked oppression, especially when moving, had a severe headache, and had increased frequency of urination at night. He vomited three or four times a day. The limbs were swollen and examination showed albumin in the urine. Under the influence of purgatives and agurin the uremic seizures gradually subsided, and the quantity of urine was augmented from 250 c.cm. to 1000 c.cm. The arterial tension was increased during the time that he took agurin.

CASE XI.—J. T., aged fifty-six years; arteriosclerosis; interstitial nephritis. Since the age of thirty-nine years the patient has had albumin in the urine, and suffered frequently from headache and a feeling of oppression. He also had to get up frequently at night to pass his water. At the time of his admission, June 12, 1902, he complained of violent headache, was unable to retain anything upon his stomach; and vomited three or four times daily. His radial arteries were hard and sinuous; there was no edema of the limbs. The patient received daily rectal injections of four grams of agurin. The urine increased to 1000 c.cm. and 1200 c.cm.; the vomiting became less frequent, and the patient could take nourishment.

CASE XII.—E. G., aged forty-three years. Lead poisoning, arteriosclerosis, interstitial nephritis. His first attack of lead colic was at thirty-two years of age. Later he suffered from rheumatism, and at forty years from pneumonia. At this time examination of the urine showed the presence of albumin. In May 1901, he commenced to experience marked oppression in breathing, headache, and suffered with severe pains over the kidneys. When admitted to the hospital the second time he had great difficulty in breathing; the limbs were edematous, and he vomited all food and medicine. Agurin was administered by rectal injection owing to the vomiting, and during the period of its administration the quantity of urine increased in amount, but fell again after it was discontinued.

CASE XIII.—A. W., fifty years old, has been a hard drinker. Cirrhosis of the liver. Since two years he has had to get up two or three times at night to pass his water, and has also suffered more or less from a feeling of oppression; at the same time he had noticed that his abdomen had increased in size. The liver and spleen were enlarged and the abdomen enormously swollen. The limbs were also edematous. At the time of his admission to the hospital he was passing 1500 c.cm. of urine daily. Under the administration af agurin, three times daily, the amount increased to only 1800 c.cm. during the next three days, and on that account the drug was discontinued.

CASE XIV.—L. H., aged thirty-eight years, suffered with an attack of pleurisy. Exploratory puncture of the chest showed the presence of a yellowish-brown liquid, containing red and white blood cells, and especially lymphocytes. The heart was enlarged. As the patient felt greatly oppressed aspiration was practiced and two liters of fluid removed. At the time of her admittance the patient was passing 500 c.cm. of urine in the twenty-four hours, a trace of albumin being present. After puncture the quantity of urine increased to 1000 c.cm. On the following day agurin was prescribed in doses of 0.5 gm. every half-hour until three grams had been taken. The quantity of urine then increased to 1200; on the next day to 1250 c.cm. The drug was continued for six days, and during this time the quantity varied between 1000 and 1200.

Although in the earlier observations we administered small doses of agurin, we are now accustomed to give it ordinarily 3 to 4 grams a day in fractional doses of 0.5 gm., at intervals of an hour. Having derived good results from rectal injections of agurin we have learned to prefer this mode of administration, for it disturbs the patient less and permits of the use of this diuretic in cases of urema with gastric disorders.

Our conclusions can be formulated as follows:

In all the patients to whom we gave agurin we have noticed an augmentation of the urine. This reached its maximum on the third or fifth day after commencing its use.

The diuretic effect persisted several days after its discontinuance.

We have observed slight increase of the arterial pressure after its administration.

The diuretic influence of agurin is variable, according to the nature of the case in which it is employed.

We have obtained the greatest increase in the quantity of urine in cases of arteriosclerosis of cardiac and renal origin. The diuretic effect has been less pronounced in cases of ascites due to cirrhosis.

The drug has been well tolerated by all our patients except one.

Its administration can be prolonged for several weeks without any disturbance.

